# Exploring the relationship of ESG score and firm value using fsQCA method: Cases of the Chinese manufacturing enterprises

**DOI:** 10.3389/fpsyg.2022.1019469

**Published:** 2022-10-11

**Authors:** Shen Zhong, Junzhu Hou, Junwei Li, Wei Gao

**Affiliations:** Harbin University of Commerce, Harbin, Heilongjiang, China

**Keywords:** ESG, firm value, fsQCA, manufacturing, China

## Abstract

The basic purpose of a business is to maximize value. With the increased expectations for companies’ social responsibility practices and sustainability, sustainability management has become a must for many companies to maximize value in the current competitive environment. Environmental, social and governance (ESG) are widely used indicators to evaluate corporate social responsibility performance. However, there is a lack of combined view on the three dimensions. This study aims to explore the sources of corporate value from the sustainability perspective. By using fsQCA (fuzzy-set qualitative comparative analysis), we proposes a configurational model consisting of environmental, social, governance, size and profitability to investigate the value-enhancing mechanisms of corporate social responsibility. The study shows that high enterprise value can be achieved through multiple paths, which can be categorized as resource-constrained type, slack resources type and good management type.

## Introduction

Maximizing value is generally considered the ultimate goal of all businesses, and this can be achieved by aligning social actions with corporate objectives to facilitate superior quality CSR programs (Malik, 2015).

Corporate social responsibility (CSR) is part of a firm’s management practice toward the public good, beyond what is required by law. To be specific, CSR is a function of a firm’s behavior toward its different stakeholders, such as communities, investors, employees, customers and suppliers, represents a firm’s discretionary multidimensional activities, which include social, ethical, environmental, and political actions (Yoon et al., 2018). In practice, ESG scores are commonly used by management consultancies and investors as a major indicator for understanding the overall CSR performance of a company. ESG primarily assesses a company’s environmental, social, governance and combines the performance of these practices (Yoon et al., 2018). A company’s social responsibility strategy is closely linked to its sustainable development plan. The aim of CSR is to build positive relationships with society and investors and to ensure the long-term profitability of the business, thereby enabling it to survive (Yoon et al., 2018). While the cost–benefit analysis of CSR has been controversial, most of the existing literature acknowledges that CSR is value-driven (Malik, 2015). This has made the CSR–valuation effect a topic of interest for many parties.

A large number of studies on the CSR-valuation effect have been published in the last few decades. However, most of the available research comes from developed economies, evidence from emerging markets is limited (Alshehhi et al., 2018). Businesses in developing countries are able to allocate fewer resources to socially responsible activities. They focusing on operational efficiency and profits rather than social values such as environmental protection, equitable distribution of wealth and community relations. This has hindered the growth of CSR in emerging markets. In this study, we address this limitation by exploring the CSR-valuation relationship and generation mechanisms in the Chinese market. In emerging markets, the CSR agenda is largely driven by governments. However, managers will only be interested in investing and disclosing their CSR if it has a positive impact on corporate value (Malik, 2015). Research suggests that the current mandatory CSR disclosure policies of some governments may not lead to improvements in corporate CSR performance unless there is a strong intrinsic motivation for companies to improve their CSR performance (Ali et al., 2017). If a positive value relevance of CSR and its generation mechanisms is verified in emerging markets, it may motivate companies to voluntarily engage in socially responsible activities and can also provide theoretical references for companies and governments to formulate business strategies and related policies, resulting in increased social welfare in emerging markets.

Further, we have considered the role of size, profitability and industry as key factors driving the CSR agenda (Ali et al., 2017). For industry, except for a few studies which showed an insignificant relationship, industry was found to be associated with CSR disclosure (Ali et al., 2017). Therefore, it is recommended that CSR research should be narrowly defined within a particular industry or context (Li and Zhang, 2010). In the Chinese context, continued rapid economic growth, driven mainly by investment in manufacturing and infrastructure, has had a serious negative impact on the environment. Legislative and administrative measures are playing a leading role in regulating the environmental behavior of enterprises (Liu and Anbumozhi, 2009). Manufacturing industry as an environmentally sensitive industry may have a stronger valuation effect of CSR than the average Chinese firm does. As for size and profitability, represent business characteristics, were found to have a significant positive correlation with CSR (Ali et al., 2017).

In addition, in the context of limited resources, there are trade-offs for companies not only between financial and non-financial performance, i.e., whether to engage CSR or not, but also between the different components of CSR (Haffar and Searcy, 2017). There is an interdependent and interactive relationship (i.e., the complementarity or reinforcement of each other) between the different components of CSR. For example, corporate governance has been shown to be positively associated with CSR (Malik, 2015). Li and Zhang (2010) found that ownership dispersion is positively associated to CSR. Using the case of Korean companies, Oh et al. (2011) found a significant positive correlation between ownership by investors concerned with corporate social activities, such as banks, pension funds and foreign investors, and a company’s CSR rating. How to make trade-offs between different CSR dimensions and how to achieve greater results with fewer resources is a question that companies need to consider. The Fuzzy-Set Qualitative Comparative Analysis (fsQCA) is considered as a powerful tool to solve the above problem (Ragin and Fiss, 2008). Based on Set Theory and Boolean Algebra Algorithm, rooted in configuration thinking, the QCA method understands organizations as clusters of interrelated structures and practices rather than sub units. It is more suitable for management involving inter-dependence and causal complexity, and has been widely used in various fields of management disciplines in recent years (Fiss, 2011). Research using fsQCA to explore the value-creating effect of CSR in the Chinese context does not yet exist. Therefore, we select 315 Chinese listed manufacturing companies, propose a configurational model consisting of environmental, social, governance, size and profitability, use QCA to investigate the value-enhancing mechanisms of corporate social responsibility.

The main results and contributions of our study can be summarized as follows. We identified four configurations of high enterprise value that can be classified as resource-constrained type, slack-resources type, and good management type. These configurations show that there are multiple paths for CSR to affect firm value. Under different conditions of corporate characteristics, the relationship between CSR and corporate value can be both positive enhancing and negative reducing effects. In addition, we find that environmental performance and social performance have substitution roles in the enhancement of corporate value. Specifically, companies of similar size and profitability can choose to improve their environmental performance or social performance to achieve high corporate performance. Further, in these configurations, the abundance of corporate resources and the level of corporate governance are very important to the value-enhancing effect of CSR. By shifting the focus from a fragmented view to the interaction among different elements, we expand and deepen the related research on how CSR affects enterprise value. Moreover, the very first use of QCA method approaching to CSR–valuation effect study enriches the existing research method box, contributes to the literature examining the configurational perspective on CSR–valuation effect.

## Literature review

### The effect CSR on the firm

Standardized, uniform and comparable CSR information is a prerequisite for CSR research. The boundaries of corporate social responsibility have been well discussed. Carroll (1979), for example, proposes four hierarchical social categories of economic, legal, ethical and discretionary responsibility; Elkington (1994) expands the traditional “bottom line” of business economics into a “triple bottom line” of social, environmental and economic considerations. However, when it comes to the specific construct of social responsibility, it is difficult to define it clearly.

The ESG concept was first introduced in the United Nations Principles of Responsible Investment, it is now widely used as an indicator by management consulting firms and investors to understand overall corporate social responsibility performance. ESG assesses a company’s environmental, social and governance practices. Specifically, environmental performance represents a company’s efforts to save energy and reduce emissions, social performance represents human rights, diversity, employee welfare, the responsibility of the product and community relations, and corporate governance performance reflects whether a company’s governance structure is sound. Although ESG is a relatively new term in the business literature, the exploration of CSR issues has been occurring for the past few decades. The cost–benefit analysis of business has always been a point of debate.

Opponents hold the view that CSR can be detrimental to corporate value. For example, Friedman (2007) famously argued that a firm’s only social responsibility is to maximize profits and that CSR should be excluded from fiduciary responsibility if it imposes unnecessary costs and leads to lower financial performance. The use of corporate resources by managers, as agents of shareholders, for purposes other than profit maximization is tantamount to misuse of money and theft. Contractarianism holds the same view. For example, Jensen (2010) claims that “multiple objectives is no objective,” social welfare is naturally maximized when the market value of each firm is maximized.

However, in the contemporary business context, the influence of these views is waning. A growing body of research shows that while CSR incurs non-essential costs, it also leads to more additional revenue, lower risk and a better corporate image. In other words, being socially responsible contributes to the growth of corporate value and long-term business. The rise of stakeholder theory has provided theoretical support for this view. Stakeholder theory suggests that the survival of an organization requires the support and approval of a wide range of stakeholders. Companies should actively adapt their behavior to gain this support and approval (Freeman, 2010). No company can succeed without meeting social responsibility targets and building partnerships with stakeholders.

In addition to stakeholder theory, other theories have also been developed to explain the value-enhancing assumptions of CSR. For example, “humane entrepreneurship” theory sees CSR as a strategic entrepreneurial gesture. The strength of a company’s entrepreneurial posture can be understood as an indicator of that company’s ability to renew and continue to seek competitive advantage in the marketplace (Parente et al., 2018). Today, there is a growing awareness of the potential impact of corporate behavior on economic development, personal and social life. This opens a window of opportunity for companies. Those companies that extend their priorities beyond their profit margin to their employees, people, environment and society earlier will have a competitive advantage over others (Parente et al., 2018).

In sum, the majority of the existing literature acknowledges the value-driven role of CSR, Margolis Joshua et al. (2018), for example, conducted a meta-analysis of 251 studies, they concluded that the impact of CSR on business performance was generally positive. However, the findings of empirical studies are still inconsistent, with positive, negative and no correlations being reported (Ali et al., 2017). One category of possible causes is the ambiguity of the definition of CSR and the differences in measurement tools (Taneja et al., 2011). Another possible reason is that there may be a time lag in the impact of CSR on corporate value. In other words, while the cost of investing in CSR is short term, the rewards are likely to be long term. As Dierickx and Cool (1989) point out, for example, strategically valuable assets, such as trust and reputation, can only be built up gradually through a series of investments. These discrete investments help firms to acquire certain stocks of assets at some point in future. Existing research also confirms that there is a time lag in the value-driven effects of CSR in specific regional and industry contexts. Using the Indian energy sector as a sample, Behl et al. (2021) observe a three-year time lag relationship between ESG and firm value. Xiong et al. (2016) found a one-year time lag relationship between ESG and firm value in the context of large Chinese construction firms. In addition, Barnett (2007) introduces the concept of “stakeholder influence capability” and argues that there is path-dependent nature in firm-stakeholder relations. Different stakeholders have different interests. Improving relationships with one group of stakeholders through one type of CSR behavior may worsen relationships with another group of stakeholders. It may even produce a net loss of overall benefit. With so many stakeholders, the trade-off between different CSR practices and stakeholders is an important and complex issue.

### A configurational approach to CSR-valuation effect

CSR can be linked to corporate values, but the different dimensions of CSR may reinforce or weaken each other. With causal complexity in minds, we propose a configurational model of CSR in driving corporate value.

#### Environmental and social performance

The existing literature provides a wealth of evidence that CSR activities can play an important role in enhancing corporate value. The benefits of CSR can be seen in many aspects of a company’s performance, such as enhanced operating efficiency, product market gains, improved employee productivity, capital market benefits, risk management, and earnings quality (Malik, 2015).

Stakeholder theory is the dominant theoretical perspective in CSR value-driven analysis (Liu and Anbumozhi, 2009). Liu and Anbumozhi (2009) classify potential stakeholders into primary and secondary stakeholders. The primary stakeholders include the shareholders, creditors, customers and suppliers. The secondary stakeholders include regulators, environmental groups and media. Protecting the interests of different types of stakeholders brings different benefits to the business (Malik, 2015). In the Chinese context, companies’ environmental protection strategies are oriented toward responding to government concerns about the environment (Liu and Anbumozhi, 2009). Businesses reduce government intervention, which can often affect business value, by using environmentally responsible activities. Unlike environmental performance, the value-driven role of corporate social performance is achieved by protecting the interests of primary stakeholders. For instance, protecting the interests of customers can create brand value, expand customer loyalty and increase sales revenue; protecting the interests of employees can improve productivity, build employer reputation and attract better personnel (Malik, 2015). The environmental and social dimensions have different stakeholder orientations, which may lead managers to make different choices when developing CSR strategies. In other words, there may be substitution roles for environmental and social dimensions in different configurations.

#### Corporate governance

How the corporate governance affects enterprise value can be investigated from the perspective of the principal agent theory. There are two problems focused by the principal agent theory: “adverse selection” and “moral hazard” (Picard, 1987). The born of modern enterprises separates ownership and control. There is a natural information gap between shareholders and management. The behavior of the agent cannot be directly observed by the principal. Due to the lack of quality and quantity of information available, the principal may not be able to choose the most suitable agent. Therefore, the adverse selection occurs (Amagoh, 2009). When the agent acts based on their own benefits instead of the benefits of the principals, and even try to get profits for themselves by harming the interests of the principals, moral hazard arises (Laffont and Martimort, 2009). The damage of the serious agency problems to the enterprise value is obvious. Based on the principal agent theory, most studies believes that a good corporate governance may not bring extra advantage and values to the company, but the neglect of corporate governance will bring additional costs and affect the ability to obtain resources.

Further, previous studies have shown an association between the level of corporate governance and CSR. For example, ownership structures can influence important company decisions such as R&D spending, innovation, capital structure, entrepreneurship, and diversification (Oh et al., 2011). Key owners (e.g., institutional owners, management owners, etc.) can propose and vote on strategic decisions through a variety of channels. Because corporate social action can be seen as an ‘investment’ (McWilliams and Siegel, 2000), it is reasonable that key owners may be involved in the company’s strategic decisions regarding social investment. Keim (1978) argues that as the ownership of a company becomes more fragmented, the demands of shareholders on the firm become diversified. In particular, investors who are concerned about corporate social activities will increase pressure on management to disclose socially responsible activities. On this basis, good corporate governance structures may have a reinforcing effect on CSR in configurations.

#### Size and profitability

Size and profitability, which represent the characteristics of a business, are the most frequently examined influencing factors of CSR in developing countries (Ali et al., 2017). There are several reasons why size and profitability should be considered when discussing the CSR-valuation effect. First, size and profitability are associated to a company’s CSR engagement. The social visibility of large, profitable companies is high. High-profile companies are often vulnerable to a variety of pressures from the media, NGOs and regulators on social and environmental issues, and they tend to respond to these issues in their disclosures in order to mitigate the pressure (Muller and Kolk, 2010). Second, size and profitability can affect a company’s CSR performance, which in turn may affect the strength of the CSR-valuation effect. On the one hand, firm size reflects a variety of attributes such as a firm’s stakeholder power, strategic posture and economic resources (Chiu and Wang, 2015), and has been shown to be positively correlated with corporate CSR performance (Muller and Kolk, 2010). For instance, (Déniz-Déniz et al., 2002) found that the number of employees had a positive effect on corporate CSP, and that larger firms had more employees. On the other hand, previous studies have shown that there is a positive correlation between profitability and CSR (Scholtens, 2008). Slack-resource theory suggests that more profitable companies have more financial resources available to undertake costly CSR initiatives, so the more profitable a company is, the better its CSR performance (Waddock and Graves, 1997; Chiu and Wang, 2015).

**Figure 1:**
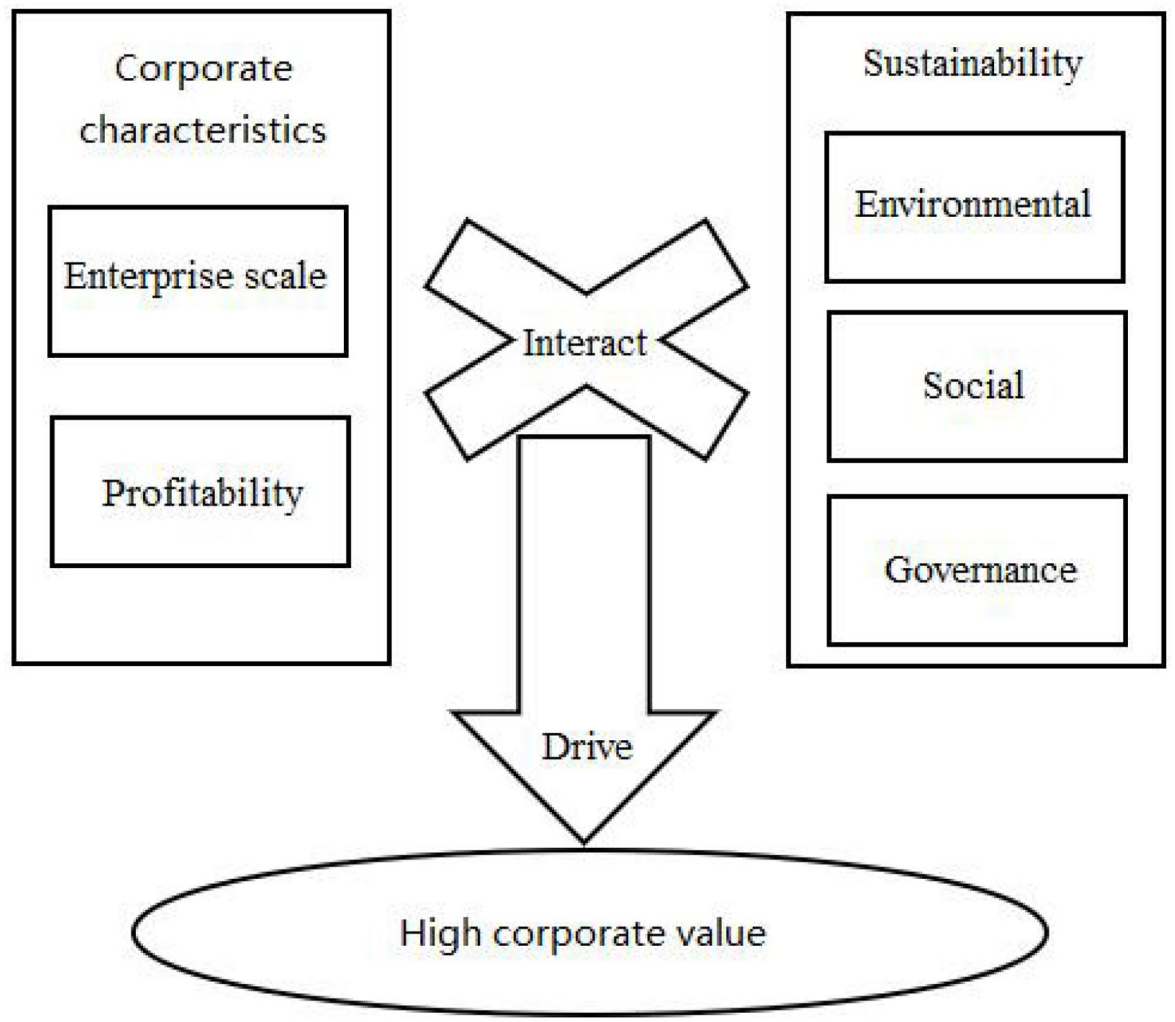
A configuration model of CSR in driving corporate value.

In sum, CSR’s role in driving value for companies is causally complex and with this in mind, we propose a configurational model consisting of five core factors: environmental, social, governance, size and profitability (see Figure 1). This configurational model should be examined in the next sections.

## Materials and methods

### Research design

QCA has its roots in political science and is now used to help management researchers determine the causal complexity of a desired organizational outcome (Fiss, 2011; Misangyi and Acharya, 2014). In this study we aim to discover different combinations of the five key components that contribute to the corporate value. By doing so, we can see the different paths to higher corporate value through CSR and understand the mechanisms behind the CSR-valuation effect.

### Sample and data collection

We have used four types of data in our study. The first is information on corporate value; the second is information on corporate social responsibility performance; the third is information on corporate governance; and the fourth is information on corporate size and profitability. We used Hexun Social Responsibility Score and data provided by China Stock Market & Accounting Research Database (CSMAR) to construct our database. The process of screening our sample can be described as follows:

1. The A-share listed companies belonged to the manufacturing industry according to the industry classification guidelines of listed companies of CSRC;
2. Excluding companies that have obvious operational problems and are not suitable for use as research cases (i.e., companies belonging to the ST and PT categories);
3. Eliminate the enterprises with missing values.

Through the three steps above, 315 observation data of China’s manufacturing listed companies are finally obtained.

### Instruments and calibration

In fsQCA method, all variables are regarded was a set. Each case has different score in different set. The process of calculating each score for different sets of each case is called calibration (Schneider and Wagemann, 2012). According to the suggestions of Ragin and Fiss (2008), combined with the characteristics and types of existing research data, we determined to use the direct calibration method to convert data into fuzzy set. Table 1 summarizes the calibration information for each condition and result.

**Table 1:** Calibration of result and conditions.

Result and conditions	Anchors		
	Full membership	Maximum ambiguity	Full nonmembership
*TOBINQ*	1	/	0
*SIZE*	1	/	0
*PROFITABILITY*	7.5	5	2.5
*ENVIRONMENTAL*	22.5	15	7.5
*SOCIAL*	30	20	10
*GOVERNANCE*	0.54	−0.24	−0.76

#### Corporate value

We use Tobin’s Q to measure corporate value. Measures of enterprise value can be either traditional accounting metrics, such as ROA and ROE, or market-based metrics, such as Tobin’s Q and PE. The true value of a business is a combination of its past performance and its current and future prospects (Malik, 2015). Market-based indicators reflect the current value of a company while also being able to predict the long-term financial performance of the company (Shank et al., 2016). As a result, research has increasingly used market-based indicators in recent years (Alshehhi et al., 2018). Therefore, in this study we use Tobin’s Q (i.e., market value/replacement cost of assets) to measure firm value. We take 1 as the threshold for calibration. When the value of Tobin’s Q is greater than 1, the market value of the enterprise is greater than the reset cost, the enterprise is developing well and the investors are confident in the enterprise, and vice versa. High enterprise value businesses are coded as 1 and non-high enterprise value businesses are coded as 0.

#### Environmental performance and social performance

We use the ESG score published by Hexun net in 2016 to measure the social responsibility performance of companies. Based on the social responsibility reports and financial reports of listed companies in China, the score contains 13 secondary indicators and 37 tertiary indicators in five areas: shareholder responsibility, employees’ responsibility, supplier, customer and consumer rights responsibility, environmental responsibility and public responsibility, which can reflect the social responsibility performance of enterprises in a more comprehensive and objective manner. This score has been increasingly used in recent years in related studies in China. There are also many other widely used external institutional scores, such as Kinder, Lydenberg and Domini (MSCI KLD Social Index), Bloomberg, and Thomson Reuters Eikon. However, there are limitations in the completeness and comprehensiveness of these data in reflecting the social responsibility performance of Chinese companies. Further, Hexun’s ESG score also compares favorably with the scores provided by other external rating agencies in China. Other external rating agencies, such as Rankins CSR Ratings (RKS), usually only disclose a company’s social responsibility rating without a specific score. There are limitations to this system in that the researcher is unable to discern differences in CSR performance between firms of the same rank. The detailed components and weights of the Hexun ESG score are shown in Table 2. We use the environmental responsibility score from Hexun to measure the environmental performance of companies. For social performance, Hexun divides it into three components: employees’ responsibility, supplier, customer and consumer rights responsibility and public responsibility, we add these three components together to get the corporate social performance score. The full scores of environmental and social scores are 30 and 40, respectively. We use the lower quartile (0.25) of the full score as the nonmembership threshold, the median (0.5) is used as the maximum ambiguity, and the upper quartile (0.75) as the full membership threshold.

**Table 2:** The components and weights of the Hexun ESG score.

Level 1	Level 2	Level 3
Shareholder responsibility (30%)	Profit (10%)	Return on net assets (2%)
		Return on total assets (2%)
		Profit from main operations (2%)
		Costs Profit (1%)
		Earnings per share (2%)
		Undistributed earnings per share (1%)
	Solvency (3%)	Current ratio (0.5%)
		Quick ratio (0.5%)
		Cash ratio (0.5%)
		Shareholder equity ratio (0.5%)
		Gearing ratio (1%)
	Returns (8%)	Dividend financing ratio (2%)
		Dividend Rate (3%)
		Dividend to distributable profit ratio (3%)
	Credits (5%)	Number of penalties imposed by the Exchange on the company and those responsible (5%)
	Innovations (4%)	R&D investment (1%)
		Technological Innovation Concept (1%)
		Number of technological innovation projects (2%)
Employees responsibility (15%)	Achievements (5%)	Per employee income (4%)
		Employee Training (1%)
	Safety (5%)	Safety checks (2%)
		Safety training (3%)
	Caring for employees (5%)	Consolation awareness (1%)
		Consolers (2%)
		Consolation money (2%)
Supplier, customer and consumer rights responsibilities (15%)	Product quality (7%)	Quality management awareness (3%)
		Quality Management System Certificate (4%)
	After-sales service (3%)	Customer Satisfaction Survey (3%)
	Integrity and reciprocity (5%)	Fair competition for suppliers (3%)
		Anti-commercial bribery training (2%)
Environmental responsibility (30%)	Environmental governance (30%)	Environmental awareness (4%)
		Environmental Management System Certification (5%)
		Amount of environmental input (7%)
		Number of discharge types (7%)
		Number of clean energy types (7%)
Social responsibility (10%)	Contribution value (10%)	Amount of public welfare donation (5%)
		Income tax to total profit ratio (5%)

Percentages in brackets represent weights.

Since the Hexun Social Responsibility Score does not disclose information on corporate governance of companies. We use principal component analysis to measure the level of corporate governance in terms of supervision, incentives, and decision making, following Ruosen et al. (2018), Zhou et al. (2020), and Hu et al. (2021). The executive compensation and executive shareholding ratio are selected to represent the incentive mechanism in corporate governance. The promotion of independent board of directors and the size of the board of directors represent the supervisory role of the board of directors. The supervisory role of the shareholding structure is expressed by the proportion of institutional shareholding and the degree of equity checks and balances (the sum of the shareholding proportion of 2–5 major shareholders/the shareholding proportion of controlling shareholders). The decision-making of the general manager is expressed by whether the chairman and the general manager are combined. The first principal component obtained from principal component analysis is used as a comprehensive index to reflect the level of corporate governance. Meanwhile, the economic significance of the index is that the higher the score of the first principal component, the better the level of corporate governance. We still use the lower quartile (0.25) of the full score as the nonmembership threshold, the median (0.5) is used as the maximum ambiguity, and the upper quartile (0.75) as the full membership threshold.

### Size and profitability

Finally, for size and profitability, we refer to the “Statistical Classification of Large, Small, Medium and Micro Enterprises (2017)” (GTZ [2017] N.O. 213) and the corporate profitability scores published by Hexun. The standards stipulate that industrial enterprises with more than 1,000 employees and business revenue of more than RMB 400 million can be classified as large enterprises, otherwise they are small, medium and micro enterprises. We code large enterprises as 1 and MSMEs as 0. For the profitability of a company, we use the profitability score as a measure, with a score out of 10. The lower quartile (0.25) of the full score is the nonmembership threshold, the median (0.5) is the maximum ambiguity, and the upper quartile (0.75) is the full membership threshold.

## Results

### Necessity conditions analysis

It is meaningful to test whether a single factor (including its non-set) is the necessary condition of the result before configuration analysis. From the perspective of the set theory, the necessary analysis of a single factor is to test whether the result set is a subset of a certain factor set. If one factor always appears in the result, the antecedent variable can be considered a necessary factor for the occurrence of the result (Ragin, 2009). Consistency is a widely used indicator to measure the necessary factors. When the consistency level exceeds 0.9, the condition is considered to be the necessary factor of the result generally. By using fsQCA 3.0, the necessity analysis results of a single factor are shown in Table 3. As can be seen from the table, none of the single antecedent variables affect the enterprise value with consistency exceeding 0.9. Therefore, no single factors form the necessary factors for results.

**Table 3:** Necessity analysis of single conditions.

Condition	High enterprise value		Condition	High enterprise value	
	Consistency	Coverage		Consistency	Coverage
*SIZE*	0.44	0.69	*SOCIAL*	0.33	0.70
*size*	0.56	0.99	*Social*	0.67	0.92
*PROFITABILITY*	0.52	0.94	*GOVERNANCE*	0.68	0.93
*Profitability*	0.48	0.74	*Governance*	0.32	0.68
*ENVIRONMENTAL*	0.27	0.67			
*Environmental*	0.73	0.91			

### Sufficient solutions

Corresponding to the necessity analysis, the configuration analysis attempts to reveal the sufficiency of different combinations of antecedent factors for the result. In other words, it reveals which combinations of antecedent factors lead to the result or not. From the perspective of set theory, it means that whether the set of different configurations composed of different antecedent factors is a subset of the result set. Different from the necessity analysis, what the sufficiency analysis needs to screen is not a single antecedent factor, but an antecedent configuration composed of antecedent factors. There are two thresholds setoff the screening of antecedent configurations: the raw consistency benchmark and the frequency benchmark. The raw consistency is used to measure the sufficiency of a specific configuration to the result. The frequency benchmark specifies the minimum numbers of cases contained in the antecedent configuration. According to the previous research and summary, there are five practical standards considered to set the raw consistency benchmark and the frequency benchmark in this paper:

1. The consistency level of sufficiency should not be lower than 0.75 (Schneider and Wagemann, 2012), but fine tune of the standard is allowed according to the actual situation, such as 0.76 (Zhang et al., 2019) and 0.8 (Cheng and Liang, 2016);
2. The configurations with the results of 0 and 1 (yes and no) in the truth table should be covered and roughly balanced (Rihoux and Ragin, 2008);
3. The set of the frequency benchmark should include at least 75% of the observed cases;
4. The minimum value of PRI (Proportional Reduction in Inconsistency) shall be equal or greater than 0.75 to reduce the potential conflict configurations;
5. Possible Simultaneous Subset Relations should be avoided. Simultaneous Subset Relations means the configuration is the sufficiency configuration of both high enterprise value and non-high enterprise value (Schneider and Wagemann, 2012).

Based on the above five practical standards, the consistency level of 0.75 and the frequency threshold of 1 is set in this paper. Since the relationship between the five antecedent factors and enterprise value in the existing research has not reached an agreed conclusion or lack of clear theoretical expectations (Schneider and Wagemann, 2012), also, there are no single condition is necessary for the outcome in the necessity analysis, “presence or absence” is chosen for this paper in the progress of generating the intermediate solution when facing the problem of which one of the five factors will lead to high enterprise. fsQCA 3.0 will output three kinds of solutions: complex solution, intermediate solution and parsimonious solution. The complexity of these solutions decreased in turn. Consistent with the existing research methods, intermediate solution and parsimonious solution are reported in this paper. Table 4 shows the configuration analysis results.

**Table 4:** Configurations strongly related to high firm value.

Antecedent condition	High firm value			
	Resource constrained	Slack resources		Good management
	1	2	3	4
Enterprise scale	⊗	●	●	●
Profitability		●	●	
Environmental	⊗		●	●
Social	⊗	●		●
Governance				●
Consistency	0.9932	0.8506	0.8271	0.7961
Raw coverage	0.5568	0.1645	0.1279	0.1212
Unique coverage	0.5568	0.0434	0.0068	0.0486
Overall solution consistency	0.9379			
Overall solution coverage	0.7768			

● = core casual condition (present). ● = peripheral casual condition (present). ⊗ = core casual condition (absent). ⊗= peripheral casual condition (absent). Blank spaces indicate “do not care.”

The consistency level of the four configurations, in both unique consistency and overall solution consistency, is higher than the acceptable minimum standard of 0.75. The consistency of overall solution is 0.94. The coverage of overall solution is 0.78, which is slightly higher than the data of QCA researches in the same organization and management field. The four configurations in Table 3 can be regarded as a combination of sufficient conditions for diversified Chinese manufacturing enterprises to get high enterprise value by using ESG method. From the perspective of each configuration (vertically), the absence of enterprise size plays a core role, while the absence of environment performance and social performance plays an auxiliary role in configuration 1 (size*environmental*social). The consistency, raw coverage and unique coverage of configuration 1 are the highest of all configurations, 0.99 and 0.56, respectively. The raw coverage and unique coverage are equal in configuration 1, which indicate that the 20 cases covered are unique coverage.

In configuration 2 (SIZE*PROFITABILITY*SOCIAL), enterprise profitability and social performance are present as core conditions. The enterprise size is present as the auxiliary factor. The consistency of configuration 2, slightly worse than the consistency of configuration 1, is 0.85 and the unique coverage is 0.04 covering 10 cases.

The enterprise profitability and the environment performance are present as core conditions while the enterprise size is present as the auxiliary factor in configuration 3(SIZE* PROFITABILITY *ENVIRONMENTAL). The consistency of this group is 0.83, which is the same as that in configuration 2. The unique coverage of configuration 3 is the lowest of three configurations covering only 8 cases.

In configuration 4 (SIZE*ENVIRONMENTAL*SOCIAL*GOVERNANCE), ESG (Environment performance, Social performance and Corporate governance) are the core factors while the enterprise size being the auxiliary factor. The consistency of configuration 4 is the lowest (0.80), and the unique coverage is 0.5 which is higher than configuration 2. Configuration 4 covers 11cases.

From the perspective of a single factor (horizontal), the existence of enterprise size appears in 3 out of 4configurations, which means this factor plays a more universal role in the impact of ESG on enterprise value, especially for manufacturing enterprises.

Finally, from the perspective among configurations (Vertical and Horizontal), there is an obvious substitution relationship between the social performance in configuration 2 and the environment performance in configuration 3, which means that these two dimensions of ESG do not need to exist at the same time to lead to the results with the remaining three factors in configuration 2 and configuration 3. According to the above complex relationships, this paper will further interpret the above four configurations by combining theories and digging in cases in the part of “Discussion.”

### Robustness test

The common methods of robustness test using QCA method mainly include: changing the qualitative anchor point of calibration data, adjusting the frequency of cases and improving the consistency threshold (Zhang et al., 2019). The robustness check in this paper uses the case frequency threshold of 2 and the consistency threshold of 0.74. Table 5 shows the configuration analysis results of the path formed by the five factors on the enterprise value adjusting the case frequency threshold and consistency threshold.

**Table 5:** Robustness test.

Antecedent condition	High firm value			
	Resource constrained	Slack resources		Good management
	1	2	3	4
Enterprise scale	⊗	●		●
Profitability		●	●	
Environmental	⊗		⊗	●
Social	⊗	●	⊗	●
Governance			⊗	●
Consistency	0.9932	0.8506	0.8787	0.7961
Raw coverage	0.5568	0.1645	0.0906	0.1212
Unique coverage	0.5033	0.0750	0.0018	0.0486
Overall solution consistency	0.9361			
Overall solution coverage	0.7717			

● = core casual condition (present). ● = peripheral casual condition (present). ⊗ = core casual condition (absent). ⊗= peripheral casual condition (absent). Blank spaces indicate “do not care.”

There are two standards of the robustness of QCA results: the set relationship state of different configurations and the difference of fitting parameters of different configurations (Schneider and Wagemann, 2012). Based on these two standards, it is easily observed that there are no significant changes in the consistency and coverage of each configuration and solution of the adjusted high enterprise value configuration, therefore, the results of this research are robust.

## Discussion

There are four paths which are identified by fsQCA for manufacturing enterprise with different characteristics to get high enterprise value using ESG. It shows that the impact of ESG on enterprise value has the same goal and multiple paths (Ragin and Fiss, 2008). According to the core conditions contained in the four paths and the explanatory logic behind them, we divide these five configurations into three types: resource constraint type, slack resources type and good management type. Among them, the resource constraint type is for MSMEs, while the slack resources type and good management type are for large enterprises. The focus of the resource constraint type is how to allocate limited resources to the optimal investment projects in order to establish and consolidate their own competitive advantage and stand firmly in the market. The large enterprises represented by slack resources and good management bear more social expectations. Improving CSR is not only a response to public pressure, but also cater to market trends, which reflects the purpose of marketing and communication more. We will discuss each of them in detail.

### Resource-constrained: The traditional strategies

In this configuration, small, unprofitable companies do not choose to invest in CSR, i.e., CSR does not play a value-driven role in the resource-constrained configuration. This is inconsistent with the value-enhancing theory proposed by Malik (2015), but corresponds to the shareholder expense theory, which emphasizes the cost aspect of socially responsible practices. It is worth noting that this configuration has the highest coverage of all configurations, which clearly highlights the different attitudes and choices of companies in emerging and developed markets when it comes to CSR. There are two possible reasons for this configuration. First, small, less profitable companies have fewer resources to allocate to social responsibility, and managers are more inclined to choose operational efficiency and profit when faced with the trade-off between financial performance and social responsibility. This confirms the claims of the slack-resources theory (Waddock and Graves, 1997). Second, according to Ali et al. (2017), firms in developing countries feel little public pressure to disclose CSR compared to developed countries, while smaller, less profitable firms have less visibility than larger, more profitable firms. As a result, smaller, less profitable companies are subject to less pressure from the media, NGOs and regulators on social and environmental issues, and companies lack the motivation to engage in CSR.

### Slack resources: Resource base

This category corroborates Ali et al.'s (2017) description that companies with high social visibility (i.e., large and profitable companies) seem to pay more attention to social and environmental issues. Because high-profile companies are often vulnerable to a variety of pressures from the media, NGOs and regulators on social and environmental issues, these pressures require high-profile companies to invest in CSR and to disclose CSR performance in order to mitigate the pressure.

It is worth noting that this category contains two configurations, where large firm size and high profitability can be combined with high environmental performance and high social performance, respectively, to achieve high enterprise value. Environmental performance and social performance show substitution roles in the configuration. This corroborates Chiu and Wang’s (2015) and Haffar and Searcy’s (2017) descriptions that there are trade-offs for firms not only in whether to engage in CSR, but also between the different dimensions of CSR. Due to limited resources, managers of a company may not give equal importance to all stakeholders. The importance of different stakeholders is determined by relationship factors, power, legitimacy and urgency. In the Chinese context, companies invest in environmental responsibility primarily to respond to regulatory requirements (Liu and Anbumozhi, 2009) and in social responsibility to protect the interests of stakeholder groups such as shareholders, employees and consumers. Since 2013, China has set the goal of industrial transformation and upgrading, and environmental protection and economic development have become the main contradictions in the current modernisation process in China (Zhang et al., 2022). As a heavily polluting industry, manufacturing is subject to stricter government regulation and greater pressure to improve environmental performance. As a result, Chinese manufacturing companies are more likely to focus their CSR efforts on improving environmental performance because the government is likely to be more powerful relative to other stakeholders, in other words, has a stronger stakeholder influence capability (Barnett, 2007).

The slack-resources configuration shows that high corporate value can be achieved by investing in either environmental or social responsibility, which gives companies more options.

### Good management: Corporate governance

The good management configuration and the slack-resources configuration represent equally large companies, which means that the companies it represents also have a high social profile and need to respond to public pressure by engaging in CSR. However, the absence of high profitability in good management configuration makes it also characterized by resource constraints, as investments in CSR are often considered to be costly. In this configuration, a high level of corporate governance compensates for a lack of profitability. The findings of Li and Zhang (2010) and Oh et al. (2011) are corroborated. Strong corporate governance mechanisms can have a positive impact on CSR. Companies with strong corporate governance mechanisms are able to improve their social responsibility performance while controlling costs and fully exploiting the value-driven role of social responsibility.

## Conclusion and managerial implications

### Conclusion

With the increasingly fierce market competitions, the pace of change makes the enterprises face unprecedented pressure. They should not only obtain competitive advantage, but also maintain it in the future. The relationship between enterprise sustainable development practice and enterprise value has been widely researched in global, but it is still difficult to reach to a consensus on the conclusion. To solve this problem, referring to the rich achievements of the previous studies, this paper constructs a configurational framework, uses the fsQCA method creatively, and uses the ESG data of 315 listed manufacturing companies in China as the research sample to explore the “joint effect” of the five influencing factors of enterprise size, enterprise profitability, environment performance, social performance and corporate governance on enterprise value, in order to explore the deep logic behind it. The conclusions are as follows:

(1) The value-driven effect of ESG has the characteristics of “multiple concurrency” and “same goal through different paths.” The value-driven effect of ESG is affected by multi-level factors, that is, multiple concurrencies. The interaction between various factors will form different paths, which means different paths lead to the same goal (Rihoux and Ragin, 2008). This paper discovers that there are four different paths for ESG practice to lead to high enterprise value, and each path is composed by different factors. This discovery is a new attempt to explain the CSR–valuation effect from an overall perspective. It is also an enrichment and supplement to the research results of the CSR–valuation effect based on contingency perspective. Therefore, the discovery promotes the change of research on the CSR–valuation effect from the contingency perspective to the overall perspective to a certain extent;
(2) The slack-resources brought by large enterprise and excellent profitability are the important factors that determine whether enterprises carry out ESG practice and whether ESG practice has a positive impact on enterprise value. It means that the enterprises with large size and strong profitability are more likely to invest surplus funds in ESG projects, in order to enhance communication with internal and external stakeholders, shape a good corporate reputation, further enhance their legitimacy and competitiveness and obtain high enterprise value. This conclusion is a confirmation of the positive impact of ESG on enterprise value and a positive response to the call to further study role of regulatory variables such as enterprise size, economy and industry type in different contexts (Alshehhi et al., 2018).
(3) There is a substitution relationship between environment dimension and social dimension in ESG. Environmental and social performance can improve different types of stakeholder relationships, enhance corporate reputation, increase shareholder returns and maximize corporate value. This is of great benefit to Chinese manufacturing enterprises to selectively invest and optimize ESG performance in different dimensions based on their own actual situation.

### Research contributions

(1) It is discovered that there is a substitution relationship between dimensions in ESG based on the situation of Chinese manufacturing enterprises. Thus, the view of stakeholder influence capability is validated. Specifically, for different companies, different stakeholders have different priorities, and companies need to choose the direction of CSR investment according to their actual situation.
(2) It is not only an expansion of the research method toolbox in this research field, but also an innovation of the epistemological basis for the CSR–valuation effect study. The vast majority of previous empirical studies in related fields have used econometric methods (Ali et al., 2017), with variables being independent of each other. We shifted our focus from single factor in previous studies to the interaction between five key factors - environmental, social, governance, profitability, and size. The configurations we derived using the QCA approach suggest that the complementarity and reinforcement between these five interdependent elements leads to high enterprise value. The configurational thinking behind QCA method is a supplement to traditional econometric methods. Introducing QCA method into the study provides a new and overall perspective for deepening the understanding and explanation of this casual complexity problem.
(3) This paper provides a new explanation for the inconsistent conclusion on CSR–valuation study. The impact of ESG practice on enterprise value is still inconclusive. For example, the negative correlation (Di Giuli and Kostovetsky, 2014), the non-correlation (Ullmann, 1985; Humphrey et al., 2012) and the positive correlation (López-Pérez et al., 2017). Our results show that the CSR–valuation effect is not a fixed linear or nonlinear relationship, nor does it all depend on the level of ESG. The interaction between the different conditions is key. Under certain boundary conditions, both trade-off perspective and slack-resources perspective can explain high levels of firm value.

### Managerial implications

1. The enterprises should act according to their own actual conditions and decision-making environment. They should not exclude ESG blindly because of its high investment, or invest in ESG blindly because of the pressure of the external environment. Among the four successful paths to achieve high enterprise value in the research result of this paper, the richness of enterprise resources and the level of corporate governance are very important for enterprises to implement ESG practice and maximize enterprise value. This means that corporate governance not only prevents the destruction of corporate value, but also enables the co-creation of value by coordinating the actors in the enterprise. Agency theory and stakeholder theory describe the role of corporate governance as balancing the interests of various stakeholders to ensure a fair and equitable distribution of wealth, thereby preventing management opportunism and expropriation. However, the results of this study show that a high level of corporate governance can itself create value for the firm. By integrating resources, managers are able to build a service ecosystem within the company, thereby increasing the adaptability and viability of the business. The professional knowledge and skills of managers are necessary in this process (Hamidi and Machold, 2020). Therefore, the Chinese enterprises should: (1) improve the corporate governance structure, expand the reserve of professionals, formulate long-term strategies for sustainable development correctly, and avoid opportunism and short-term behaviors; (2) incorporate ESG into a part of the enterprise’s business strategy formally, regard ESG as a business mode and investment method, rather than a simple capital outflow behavior, so as to allocate resources for it reasonably.
2. The government should continue to vigorously promote ESG related policies and expand the influence and binding force of ESG on enterprise behaviors. It is discovered that the path with the highest coverage and consistency of solutions is still the path to implement traditional business strategies under resource constraints even though there is a path to improve stakeholders’ relations and achieve high enterprise value by using ESG practicing. In other words, ESG practice is still the “privilege” of large enterprises in the results presented in this study, which proves that the popularity and practice of ESG in Chinese enterprises need to be improved. This paper puts forward the following opinions and suggestions: (1) Establish and improve the ESG disclosure system and unify the ESG evaluation standards gradually. Although many external rating agencies in China have issued ESG rating reports recently, there is a lack of communication and coordination among various institutions. The reports have different emphases, different calculation standards and different rating results, which makes it difficult for investors to identify ESG investment. This restricts the development of ESG investment to a certain extent in China. (2) Formulate certain reward and punishment strategies according to the practice of enterprise ESG, such as giving high ESG performance enterprises certain credit concessions and tax relief, establishing a “black list” of enterprises with poor ESG performance, increasing their “pollution tax” burden, guiding banks to reduce their loan amount or increase their loan interest rate, guiding enterprise to strengthen the performance of ESG at relevant levels on its own initiative and disclose ESG related information actively.

## Limitations and future scope

Same as other studies, this paper has some deficiencies inevitably. The shortcomings of this paper can be summarized into the following two points: (1) from the aspect of research design: firstly, this study can only select 315 manufacturing enterprises from more than 1,000 manufacturing enterprises due to the integrity of ESG information disclosure, and these 315 manufacturing enterprises cannot include all manufacturing industries. Secondly, although this paper has tried to include important influencing factors by limiting Chinese manufacturing enterprises and including enterprise size and profitability, due to the limited diversity of may be caused by QCA method, it is inevitable that some regulatory variables are not included in the configurational framework. For example, the ownership nature and debt level of enterprises. (2) From the aspect of research method selection, the study uses the static QCA method. The progress of dynamic QCA method is relatively behind. Although there are dynamic QCA methods such as TQCA and TSQCA, it cannot achieve the purpose of analyzing how the co-evolution of multiple trajectories affects the result carefully. Enterprise ESG investment brings more long-term benefits to enterprises. Due to the relatively backward development of ESG in China, investors may have a certain time lag in the value of evaluation of high ESG level enterprises (Behl et al., 2021). Under this situation, if we can explore the driving mechanism of enterprise value under the ESG framework from the perspective of time series, we may get more accurate and practical results.

For future research directions, sustainability and digital transformation may be an interesting topic with the advent of the Fourth Industrial Revolution. Digital transformation is considered to be one of the best commercialisation practices nowadays as it ensures that companies remain competitive in a rapidly changing business environment (Ponsignon et al., 2019). Existing research has clearly expressed a positive correlation between digital transformation and sustainability. Many companies have integrated sustainability strategies into their digital transformation roadmap, adopting cleaner and more sustainable production processes. Such sustainable production systems improve profitability, reduce operating costs and increase employee safety and ultimately lead to increased corporate value (Rosário and Dias, 2022). The interconnectedness and interdependence of digital transformation and sustainability in enhancing enterprise value is worth exploring. In other words, how can the elements of digital transformation and sustainability be configured to achieve high enterprise value? Future research could develop a configurational model to explore the mechanisms by which sustainability practices impact on enterprise value under conditions of digital transformation.

## Data availability statement

This data can be found here: The data underlying the results presented in the study are available from CSMAR database and Hexun database. https://www.gtarsc.com, https://www.hexun.com.

## Author contributions

SZ: conceptualization and methodology. JH: data curation and writing-original draft preparation. JL: supervision and writing-reviewing. WG: software. All authors contributed to the article and approved the submitted version.

## Funding

This study was funded by the Heilongjiang Philosophy and Social Sciences Project (21JYE396).

## Conflict of interest

The authors declare that the research was conducted in the absence of any commercial or financial relationships that could be construed as a potential conflict of interest.

## Publisher’s note

All claims expressed in this article are solely those of the authors and do not necessarily represent those of their affiliated organizations, or those of the publisher, the editors and the reviewers. Any product that may be evaluated in this article, or claim that may be made by its manufacturer, is not guaranteed or endorsed by the publisher.
